# Automated Platforms in *C. elegans* Research: Integration of Microfluidics, Robotics, and Artificial Intelligence

**DOI:** 10.3390/mi16101138

**Published:** 2025-10-01

**Authors:** Tasnuva Binte Mahbub, Parsa Safaeian, Salman Sohrabi

**Affiliations:** Department of Bioengineering, University of Texas at Arlington, Arlington, TX 76019, USA; tasnuva.mahbub@uta.edu (T.B.M.); parsa.safaeian@uta.edu (P.S.)

**Keywords:** *C. elegans*, automation platforms, microfluidics, micromanipulation techniques, imaging, assay automation, high-throughput screening, robotics, artificial intelligence

## Abstract

*Caenorhabditis elegans* is one of the most extensively studied model organisms in biology. Its advantageous features, including genetic homology with humans, conservation of disease pathways, transparency, short lifespan, small size and ease of maintenance have established it as a powerful system for research in aging, genetics, molecular biology, disease modeling and drug discovery. However, traditional methods for worm handling, culturing, scoring and imaging are labor-intensive, low throughput, time consuming, susceptible to operator variability and environmental influences. Addressing these challenges, recent years have seen rapid innovation spanning microfluidics, robotics, imaging platforms and AI-driven analysis in *C. elegans*-based research. Advances include micromanipulation devices, robotic microinjection systems, automated worm assays and high-throughput screening platforms. In this review, we first summarize foundational developments prior to 2020 that shaped the field, then highlight breakthroughs from the past five years that address key limitations in throughput, reproducibility and scalability. Finally, we discuss ongoing challenges and future directions for integrating these technologies into next-generation automated *C. elegans* research.

## 1. Introduction

Technological platforms such as microfluidics, robotics and artificial intelligence (AI) have become fundamental in contemporary biological research due to the ability to precisely manipulate fluids, reagents and biological samples ranging from unicellular to multicellular organisms in miniaturized or automated environments. Among them, microfluidic systems are typically built using transparent and inert biocompatible materials like PDMS or hydrogel, that allow precise fluid flow, limited reagent use, real-time observation under microscopes, controlled reagent and stimuli delivery while maintaining compatibility with high-resolution imaging tools [1,2,3,4,5]. The ease of soft lithographic fabrication, along with integration of microvalves, micropumps and automated controllers to these devices, offers reduced experimental variability and large-scale multiplexing capacity [1,2,3,4]. Meanwhile, robotic systems, AI-based analysis pipelines and micro-electromechanical platforms have expanded experimental throughput, real-time feedback control, enhanced reproducibility and reduced manual workload [3,4,6]. Although applied to diverse model systems like cell lines, Drosophila, zebrafish etc., these platforms are most widely used in studies involving the nematode, Caenorhabditis elegans (*C. elegans*), whose body dimensions and physiological characteristics align exceptionally well with the microscale features.

*C. elegans* was first established as a genetic model by Sydney Brenner in the 1960s and has now become a cornerstone of biomedical and genetic research [6,7]. This soil-dwelling nematode is small (~1–1.2 mm), with a life cycle of 3 days and a typical lifespan of 2–3 weeks depending on temperature and living conditions, making it highly suitable for rapid experimentation [8,9,10]. *C. elegans*, having a fully sequenced genome and completely mapped nervous system, revealed that around 40% of its genes have human homologs, consists of 302 neurons and has conserved signaling pathways [2,5]. Its transparency at all life stages permits real-time high-resolution in vivo imaging, including the use of fluorescent markers to visualize gene expression and neuronal activity [4,11]. All these combined have enabled disease modeling (e.g., Alzheimer’s, Parkinson’s and Huntington’s disease), neurobiological and cognition studies in *C. elegans* [9,10,12]. Together, these features make *C. elegans* a powerful and manipulatable in vivo model organism for studying fundamental biological processes and complex human pathologies in a tractable and scalable manner.

While worm-based studies have been so mainstream, traditional manual approaches like Petri dish assays or glue-based immobilization are often slow, tedious, labor-intensive, provoke side effects and lack reproducibility, especially in high-throughput settings. The development of automation platforms has significantly transformed these worm assays by offering precise stimuli and reagent delivery, high-throughput and reproducibility, a controlled experimental environment, eliminating chemical anesthetics or constant human intervention. Because of their affordability, adaptability and biological compatibility, microfluidic platforms continue to lead the way. However, pairing them with robotics, AI and optoelectronics has opened a new dimension in worm assays like robotic microinjection, automated sorting, closed-loop control, multimodal data acquisition and real-time observation [10,13,14]. This has fast-paced biological discovery while promising longitudinal phenotyping, accuracy and scalability.

This review seeks to offer an updated perspective by emphasizing recent innovations from the past five years (2020–2025), covering not only microfluidic systems but also a wide range of engineered platforms for micromanipulation (immobilization, sorting, microinjection and microsurgery), imaging, assay automation and high-throughput screening. Moreover, this review focuses on how these devices have addressed longstanding drawbacks, what challenges they still carry and how future systems might integrate across technologies to advance the next generation of scalable, high-content *C. elegans* experimentation.

## 2. Micromanipulation Techniques

Micromanipulation techniques like immobilizing, sorting and injecting worms require high expertise with worm handling, as handling error can often exert stress on animals and introduce operator-dependent variability in experimental results. Microscale tools have made it simpler and more efficient to work with *C. elegans* in a controlled and consistent manner by addressing these issues and improving the data quality and repeatability. Researchers can now more precisely mimic the experiments with no user-induced variability and track the same animal over time. Recent developments that have automated the core micromanipulation techniques are highlighted in this section.

### 2.1. Immobilization

Due to the dynamic nature of *C. elegans*, they are often required to be immobilized. Traditional immobilization methods involve anesthetics and adhesives, which often compromise worm viability, induce toxicity and side effects, trigger stress responses and reduce experimental throughput. Throughout the years, many studies have come up with new strategies for worm immobilization, addressing the previous limitations, that are both reversible and physiologically gentle. These automating devices, mostly microfluidic, have enabled precise and repeatable immobilization through a variety of mechanisms, including mechanical trapping, thermal modulation, chemical exposures, optical or photothermal control and acoustic actuation. Some notable previous works are presented below.

#### 2.1.1. Advances in Immobilization Techniques up to 2020

Focusing on mechanical interventions for immobilization, trapping structures such as tapered channels and fixed-geometry clamp arrays enabled physical confinement of worms without chemical anesthetics [2,9]. Using 128 wedge-shaped microchannels in a microfluidic array, Hulme et al. [15] immobilized more than 100 worms in parallel, and similarly, Allen et al. [16] used these tapered channels to immobilize animals for targeted neuronal laser ablation. Improving this tapered channel design, Berger et al. [17], conducted vulva-aligned immobilization using on-chip hydraulic valves, enabling high-resolution imaging while allowing egg laying. A couple of PDMS-based microfluidic platforms fabricated by Guo et al. [18] and Zeng et al. [19] and further modified by Mondal et al. [20] and Gilleland et al. [21], used micro valve-actuated flexible membranes for compressive immobilization with reversible fixation, allowing subcellular imaging, axotomy, and microsurgery. More recently, in compressive immobilization, Martinez et al. [22] and Keil et al. [23] achieved periodic immobilization, long-term worm observation, and fluorescence imaging of individual worms at spatiotemporal resolution, all of which enabled temporal tracking of dynamic cellular processes. Shifting to thermal modulation strategies, the sol–gel transition of Pluronic F127 was the most noteworthy one. Krajniak et al. [24] and Aubry et al. [25] used this for temperature shifting to perform reversible worm immobilization, allowing high-resolution imaging and sorting for worm developmental analysis. This strategy was expanded by Gijs et al. [26], who used hydrogel compression aided by microbeads for immobilization, that monitored protein aggregation and mitochondrial morphology. In addition to this approach, Chuang et al. [27] presented a unique laser heating technique. They created a microfluidic device called “addressable light-induced heat knockdown (ALINK),” which uses laser-induced heating in a liquid medium to immobilize worms in less than 10 s, enabling morphological analysis. Concurrently, Cornaglia et al. [28] created a hybrid platform for dynamic monitoring of gene expression and protein aggregation in neurodegenerative diseases by combining on-chip worm culture, embryo incubation, reversible immobilization and high-resolution imaging. Leveraging towards chemical immobilization, Chokshi et al. [29] introduced a CO_2_ microenvironment where CO_2_ diffusion into deformable PDSM layers creates compressive immobilization.

These methods collectively show how microfluidic immobilization techniques have developed over time to improve worm viability, subcellular resolution imaging, and throughput in a variety of biological assays. However, the techniques still remain constrained by low precision early-stage immobilization, disruption to natural worm behavior with repetitive imaging and preprocessing of worms before immobilization. Recent innovations in immobilization techniques have been responding to these drawbacks, while conserving behavioral integrity.

#### 2.1.2. Recent Advances in Immobilization Techniques (2020–Present)

In the past five years, three notable works have been introduced with unique approaches. Firstly, Sridhar et al. [30] introduced a contactless method for momentary immobilization of worms using a combined thermal and acoustic pressure modulation. They developed a surface acoustic wave (SAW)-based microfluidic platform that offered non-invasive immobilization for 30 s. The PDMS-based device consisted of a fluidic chamber incorporating a lithium niobate substrate and an interdigital transducer (IDT) that generated traveling SAWs, which in turn exerted acoustic pressure to gently immobilize worms. Repeated immobilization cycles on the same animal caused partially reversible physical deformation, but survival assay for over 72 h and heat-shock assay proved to cause no long-term harm in worms due to immobilization. Moreover, the cycles require sufficient breaks in between for cooling down to room temperature, as the acoustic pressure generates heat. The first correlation between glutamatergic synaptic receptor dynamics and swimming in aging worms was established, linking synaptic receptor regulation with age-induced locomotory decline, by conducting a longitudinal study in this device. The device holds notable potential for repetitive assays and imaging for developmental, behavioral and morphological analysis.

In contrast to the SAW device requiring heat and acoustic pressure, Wang et al. [31] leveraged a cooling strategy to immobilize worms directly on agar plates in a non-contact method, bypassing microfluidics. Their cooling apparatus, named ‘Copli: cold plate immobilization’, consists of a cooling stage for placing agar plates, a Peltier heat pump to remove heat from agar plates and a liquid cooler to discard the heat received from Peltier. Worms cultivated at 20 °C were immobilized at 6 °C by reversibly inhibiting neuromuscular function and imaged with a submicron-resolution microscope. However, worms required a long recovery period and gentle touch to regain motion, even after returning to 20 °C. While ideal for slow cooling and high-throughput fixed-point imaging, the platform is not suitable for high-intensity fluorescence imaging, as it triggers motion and variability of optimized immobilization temperature. Despite these limitations, Copli demonstrated how lifespan is not affected by cooling and the transcription factor CREB/crh-1 drives lesion-conditioned neuronal regeneration. 

**Figure 1:**
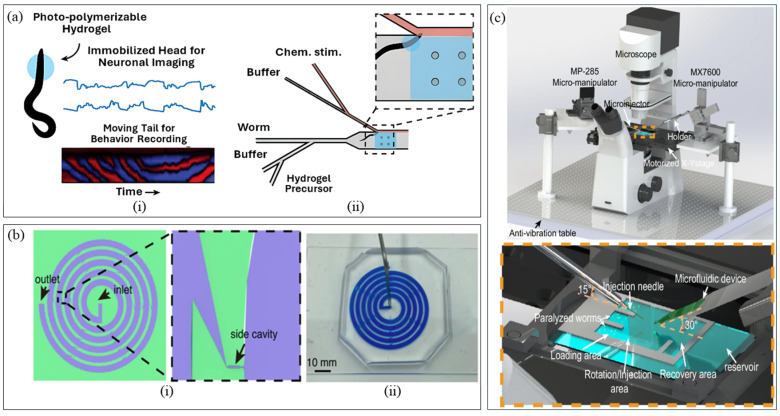
Devices for *C. elegans* Micromanipulation. (**a**) Immobilization platform (Reproduced with permission from [Hyun Jee Lee et al. [32]], [Reaction Chemistry & Engineering]; published by [Royal Society of Chemistry (RSC)], [2023]); (**b**) Sorting Machine (Reproduced with permission from [Peng Pan et al. [41]], [Microsystems & Nanoengineering]; published by [Springer Nature], [2023]); (**c**) Microinjection platform (Reproduced with permission from [Peng Pan et al. [53]], [Nature Communications]; published by [Springer Nature], [2024]) (Pan et al. [53]). The left part of panel (**a**) shows the photo-polymerizable hydrogel for worm head immobilization, retaining tail motion, while the right part portrays the microfluidic platform for repetitive neuronal imaging and chemical delivery. Panel (**b**) depicts the schematic of the microfluidic device on the left and the real image of the device on the right. Panel (**c**) has the entire microinjection setup at the top, while the bottom side is the detailed image of the individual components of microinjection.

Shifting the focus to only neural imaging, Lee et al. [32] devised a hybrid platform (Figure 1a) integrating hydrogel photopolymerization and microfluidics for immobilization of the worm’s head while allowing the tail to move. The UV-induced hydrogel polymerization occurs inside a PDMS-based microfluidic platform in two steps: initial polymerization for directing and positioning the anterior region of worm’s head, and the second step for immobilizing. This configuration enabled repetitive high-resolution fluorescence imaging of neural activity due to chemical stimulation to worm’s nose while allowing tail movement for simultaneous behavioral tracking. The method offers a concurrent correlation of neuronal dynamics with behavior, high-throughput neural imaging, scope for both full-body and worm head immobilization. But concerns regarding possible phototoxicity due to localized UV exposure upon repeated use persist and might be addressed by investigating thermoresponsive or visible-light crosslinkable hydrogels in future versions. 

Taken together, these recent advances address major limitations such as reliance on physical confinement or chemical agents, inability to perform multiplexed analyses and restricted throughput, but they still await AI integration for greater automation and robustness. Importantly, the choice of immobilization technique depends entirely on the specific requirements of the experiment and the overall study design. For example, for neuronal studies, immobilizing only the head part is enough, whereas for morphological analysis would preferably require whole body immobilization. Moreover, immobilization by cooling is more beneficial for neuronal studies as it is the only resort to halt pharynx activity, whereas for morphological or behavioral analysis other immobilization techniques suit well too.

### 2.2. Sorting

Worm sorting is a fundamental step for high-throughput screening and sophisticated research like aging, behavioral, neurobiological and genetic studies. Depending on the type of research being conducted, *C. elegans* are often required to be sorted in high throughput based on sex, developmental stage, size, phenotype, neuronal or behavioral traits.

#### 2.2.1. Key Advances in Sorting Strategies up to 2020

Commercial non-microfluidic devices like the COPAS and BioSorter offer high-throughput fluorescence-based sorting but are limited by insufficient subcellular resolution, operational complexity and maintenance cost [5,13,33]. Responding to these limitations, microfluidic platforms have emerged, offering various sorting strategies. These strategies fall broadly into three categories: passive, field-based and actuation-based. Passive sorting relies on channel geometry or hydrodynamic resistance to separate worms without external forces [9], like Solvas et al. [34] using interconnected bifurcated flow channel structure termed as ‘smart maze’ for hydrodynamic movement disruption and Ai et al. [35] using micropillar array as sieve for size-based sorting. Field-based sorting employs electrotaxis or surface acoustic waves to isolate worms, like Han et al. [36] using electrotaxis to sort worms based on swimming rate while Rezai et al. [37] sorting worms based on worm paralysis in varying electric field intensity, with difficulty in sorting between immediate developmental stages (e.g., L2 vs. L3). Actuation-based sorters include mechanical components like valves and controllers, like the one developed by Dong et al. [38] using pressure-driven membrane for worm separation based on movement restriction under different flow pressures; or by Rohde et al. [39] employing low-pressure-controlled microvalves and suction for phenotypic worm sorting.

Despite these advances, these systems face trade-offs like incorporating multi-feature sorting capacity and distinguishing between immediate developmental stages. These constraints have driven the development of recent innovations addressing these critical gaps. Such notable platforms from the last five years have been introduced here.

#### 2.2.2. Recent Progress in Sorting Strategies (2020–Present)

Overcoming the constraints of limited subcellular resolution and a narrow set of sortable features in previous COPAS Infinity models, the recent COPAS Infinity model [40] introduced a multi-feature, larger operational size range and high-throughput solution. The system can now analyze up to 31 optical parameters, including fluorescence intensity and optical scattering, allowing reliable sorting even for worms expressing weak GFP or subtle fluorescent reporters. Its expanded size range of 2–1500 microns in diameter allows precise sorting even between immediate developmental stages of worms. Worm viability is preserved through an optimized fluidic path and air-puff sorting mechanism. Additionally, the integration of LP Sampler has automated sorting from standard multiwell-plates, further enhancing throughput and reducing manual handling. While these advances address several major limitations of earlier models, cost and maintenance still remains a vital issue.

To study embryonic development dynamics in *C. elegans*, Pan et al. [41] resorted to passive sorting and trapping of embryos, integrated with long-term imaging. They presented a single-layered spiral microfluidic device (Figure 1b) that used Dean drag and lift forces within spiral microchannels to achieve size-based separation. Worms of all developmental stages flowed through the spiral channel, having embryos directed towards the outer wall, while larger larvae and adults flowed towards the inner wall. Following separation, passive hydrodynamic trapping captured single embryos in each side cavity with ~85% efficiency, allowing extended high-resolution imaging. Using this approach, the authors observed flow rates and osmotic stress affecting the developmental rate of embryos. This platform lacks an integrated temperature control, which may render embryos more susceptible to stress during prolonged imaging. Taking cost-effective access to microfluidic sorting, Peters et al. [42] fabricated a 3D-printed double-layered microfluidic platform. Unlike conventional systems, worms are separated by size at an 11.7 µL/min flow rate and 2.3 worms/min with 83% sorting purity. Demonstrated with 10 anesthetized *C. elegans*, the system was capable of sorting only two major developmental stages, embryos and fully grown adults, under manually controlled syringe-driven flow. While the device simplifies the fabrication process and lowers costs, it faces challenges in sorting multiple developmental stages, sorting parameters and rate, and lacks integrated imaging for phenotype confirmation. Potential improvements could include incorporating optical modules for real-time detection, optimizing channel geometries and dimensions to sort all developmental stages, introducing multi-channel arrays coupled with automated valves and pumps to enhance sorting rate and throughput under controlled flow conditions.

Collectively, these platforms address distinct limitations in *C. elegans* research, from the high-throughput but costly COPAS Infinity to Pan et al.’s [41] device for embryonic sorting and longitudinal imaging, and Peters et al.’s [42] 3D printed system offering a cost-effective entry into microfluidics. While this micromanipulation technique has advanced significantly, it has yet to explore AI integration in this sector.

### 2.3. Microinjection and Microsurgery

Microsurgery and microinjection are the foundational techniques for in vivo *C. elegans* studies, like intercellular communication, reproductive, neuronal, or genetic studies. These techniques provide precise neuronal and cellular ablation, delivery of genetic material, dyes, proteins, or chemical reagents at the cellular and subcellular level [5,43,44]. Traditionally, this technique is performed by manually anesthetizing worms on a glass coverslip with agarose pads and delivering reagents using a micromanipulator and glass microneedle. The method being highly skill-intensive, time-consuming and human error-prone, limits throughput and precision significantly [5,45]. For instance, early manual microsurgery and microinjection methods were performed by Avery and Horvitz [46] for neuronal laser ablation and germline transformation by Mello and Fire’s seminal protocol [45], respectively.

#### 2.3.1. Technological Advances in Microinjection and Microsurgery up to 2020

Responding to these challenges of manual approaches, some notable microfluidic systems were introduced throughout the years that have automated microinjection and microsurgery via laser, device geometry, or active elements like valves. For instance, to perform single-animal femtosecond laser microsurgery, Guo et al. [18] and Zeng et al. [19] designed valve-based compression systems for trapping worms, whereas Gilleland et al. [21] introduced elastomeric flexible layers for mechanical worm trapping. Aiming for improved targeting precision, Chung and Lu [47] developed a fully automated, high-throughput cell microsurgery system combining cooling-based worm immobilization, real-time image-guided neuron targeting, and automated laser ablation. For microinjection, both Zhao et al. [48] and Song et al. [49] used suction-based immobilization, where former one attempted manual injection and the latter one automated it by robotic image-guided needle operation and micromanipulator-controlled injection. These and many more systems, over time, have minimized manual handling and animal stress while enabling high-throughput micro interventions with significantly reduced time requirements. Nevertheless, these microinjection and microsurgery techniques have been limited by their narrow application across different worm types and morphologies, variability in needle placement precision and only partial automation; factors that continue to drive innovations aimed at advancing this field.

#### 2.3.2. Current Advances in Microinjection and Microsurgery Platforms (2020–Present)

Targeting precise microinjection alignment without an expensive multi-degree-of-freedom (DOF) micromanipulator, Ghaemi et al. [50] designed a mechanically driven microfluidic microinjector that combined a PDMS chip with passive immobilization and a single DOF micro positioner. Worms were passively immobilized in ~55 μm-wide tapered channels, followed by precise positioning of needle into the worm gonad via a single DOF micromanipulator and pressure-actuated reagent delivery. The device cut the entire microinjection process time from 10 s of min/worm to ~30 s/worm, improving gonadal delivery success from 30% to 63%. However, throughput remained limited to one worm at a time, had occasional needle misalignments and required manual worm loading. On a different approach, Gibney et al. [51] proposed using a moistened fine-tipped paintbrush for transferring and positioning worms on agarose pads, replacing the conventional metal picks. The paintbrush reported picking 20 worms at a time with enhanced worm longevity and reduced mechanical stress during transfer, followed by lateral alignment of the gonad of at least 15 worms in <1 min. This simple process increased worm survival by 62%, tripled throughput (from ~1–2 worms/min in traditional picks to ~3 worms/min in paintbrush) and reduced operator error, particularly for beginners. Lockery et al. [52] developed the “Poker Chip,” a PDMS-based microfluidic platform for microinjection, featuring a tight-fitting immobilization channel with a thin elastomeric side wall serving as the injection site. Worms were pressure loaded via a handheld syringe, which then naturally aligned laterally with the gonad, while the pipette penetrated through the PDMS septum for reagent delivery and resealed upon withdrawal, allowing reuse. The device offers a potential replacement for manual agar-pad germline transgenesis, achieving comparable survival and transformation rates to traditional methods (no significant difference statistically). Although successful injections still relied heavily on operator skill, as the pipette insertion angle had to be carefully matched to microchannel dimensions, authors recommended 1–2 practice rounds (~5 min) for reliable performance. Most recently, Pan et al. [53] engineered a microinjection platform (Figure 1c) integrating microfluidics with robotics to improve *C. elegans* transgenesis, overcoming the limitation of manually setting pipette insertion angle. The system combines pressure-actuated immobilization, hydrodynamic rotational alignment, visually guided closed-loop PID control for automated pipette insertion and solution delivery via a pneumatic microinjector. Worms were anesthetized, loaded into a microfluidic device by negative pressure, oriented by hydrodynamic rotation in the injection site for precise gonad alignment, immobilized and injected at a rate of 44.5 s per worm across wild type (almost thrice as that of manual injection rate of ~2–2.7 min/worm by experts), mutant and curled up anesthetized strains. While this platform discovered the underlying regulatory RNA-binding protein, TDP-1, for the alternative splicing of zoo-1 mRNA, it still required prior worm anesthetization.

Moving on to microsurgery, Harreguy et al. [54] introduced an Ytterbium-doped fiber femtosecond laser system for *C. elegans* neuronal microsurgery, enabling deep (>100 µm) and highly precise axotomy with minimal collateral damage. The system generates ~400 fs pulses at 1030 nm, delivered through a beam expander and focused via a microscope objective, with only 20% of the laser output sufficient for effective neuronal ablation. Compared with nanosecond solid state lasers and femtosecond Ti/Sapphire, it provided smaller lesion size (~12–14 µm, ~1.6 µm, and ~1.3 µm lesions, respectively), proved more cost-effective, required less system repositioning and incorporated software-based pulse picking. This system allowed tunable lesion size along with bundle-level ablation without peripheral damage and yielded comparable neuronal regeneration outcomes to the other systems. In the upcoming year, Fouad et al. [55] introduced an infrared-based (1480 nm) thermal laser ablation system as an alternative to short-pulsed lasers, ablating larger areas not achievable with previous traditional plasma-based systems. Traditional plasma laser required many pulses for 2–4 µm-wide ventral nerve cord (VNC) with cuticle rupture risk, whereas this system improved that to entire VNC ablation without any injury with just 10–20 pulses. A pulse generator produced tunable infrared pulses, focused through a microscope objective onto the targeted ablation site in worms. Using this system, they performed microsurgery on the VNC of acr-2(gf) mutants and demonstrated that convulsions arise independently from multiple loci rather than a single origin. The post-microsurgery recovery of nerves was unknown in this work, which was addressed by the recent work of Zhao et al. [56]. They reported a femtosecond laser system for micrometer-precision microdissection of a single regenerating neuron in living worms, enabling single-cell RNA sequencing. Their method involved encasing the target neuron by ablating surrounding tissue to form a 3D shell, low-energy laser delivery to slightly burn the tissue, followed by neuronal ablation while maintaining a 10–20 µm safe distance to prevent cellular content loss. After a short sealing period, a 2 µm cuticle section was ablated to release the dissected neuron via internal pressure. This approach increased cell yield to 32% from 0.5%, enabled identification of axon regrowth-related 226 candidate genes, explained why some neurons succeed or fail regeneration by dividing those genes into six clusters based on specific biological roles, alleviated transcriptional artifacts and supported phenotype–genotype mapping. Although limited by low throughput (~five neurons/hour) and high cost, further automation holds promise for significantly improving throughput and scalability.

In summary, recent innovations in *C. elegans* microsurgery and microinjection span from microfluidic chips to robotic platforms and advanced optical systems. As these tools become increasingly precise and targeted, each addressing specific drawbacks, they also introduce greater technical complexity, necessitating specialized training and expertise. 

## 3. Worm Imaging

Worm imaging is a crucial component of *C. elegans* research, providing insights into neurological, physiological and morphological processes. Traditional imaging relied on anesthetic-based immobilization for static snapshots, but recent automated platforms enable continuous, high-resolution and rapid capture of dynamic physiological events using advanced immobilization methods, as discussed earlier. Chronis et al. [57] were among the first to implement microfluidics for in vivo, real-time neuronal and behavioral calcium imaging using GCaMP, to reveal correlations between interneuron activity and locomotion, and between chemosensory neurons and osmotic stimuli. Shi et al. [58] extended the neuronal imaging approach with high-throughput fluorescence microscopy platforms integrating droplet microfluidics to study neurotoxic responses to neurotoxin agents. Krajniak et al. [24], Wen et al. [59], and Saberi-Bosari et al. [60] integrated perfusion and flow control with fluorescence microscopy for long-term, repeated phenotypic imaging under various physiological conditions, aging and polydatin chemical agent, respectively. Zhu et al. [61] developed a microfluidic chip for parallel fluorescence imaging of metabolic disorder biomarkers in hyperglycemic worms over their lifespan, enabling real-time single-animal tracking at single-animal resolution. Similarly, Cornaglia et al. [28] introduced another microfluidic platform that allowed parallel, multi-dimensional imaging at single-embryo resolution under controlled conditions to study mitogenesis in developing embryos. In a slightly different approach, Hwang et al. [62] employed optogenetics with microfluidics to trigger muscle contraction for studying sarcomere phenotypic analysis, offering a new route to light-induced cellular processes in live animals. Collectively, these platforms demonstrate how imaging has evolved from taking static snapshots to fully integrated systems for dynamic worm analysis. Yet, challenges remained in the trade-off between speed and resolution, throughput, full automation and scalability, driving new generation imaging innovations.

### 3.1. Emerging Trends in Worm Imaging (2020–Present)

#### 3.1.1. Microfluidic-Based Imaging Platforms

Neuronal imaging in *C. elegans* is often limited by one-sided immobilization, making bilateral neurons difficult to capture. Chung et al.’s [63] addressed this with a rotatable side-view microfluidic device that enabled calcium imaging of bilateral neurons on a single focal plane using an epifluorescence microscope. In this setup, worms were introduced into the trapping channel via syringe injection rather than direct picking, while the entire setup was rotated for imaging. Using this system, they studied ASE and ASH sensory neuron responses to varying NaCl concentrations and found them to be random. Shifting to dynamic subcellular imaging, Mondal et al. [64] fabricated a PDMS-based microfluidic platform for long-term culture and anesthetic-free immobilization, facilitating repeated high-resolution imaging of the same worm for over 36 h. Their study revealed that mitochondrial additions occur randomly along neuronal processes via active transport when inter-mitochondrial distance exceeds ~24 μm. Both platforms, however, remain limited to single-worm imaging, restricting throughput. The rotatable device further required manual injection, head-first orientation and constrained natural displacements, adding labor and risk of worm damage. Optimizing the microchannel design for passive immobilization and worm head orientation for the rotatable microfluidic device, and multiple worm housing with simultaneous imaging for both devices could be considered as future work.

**Figure 2:**
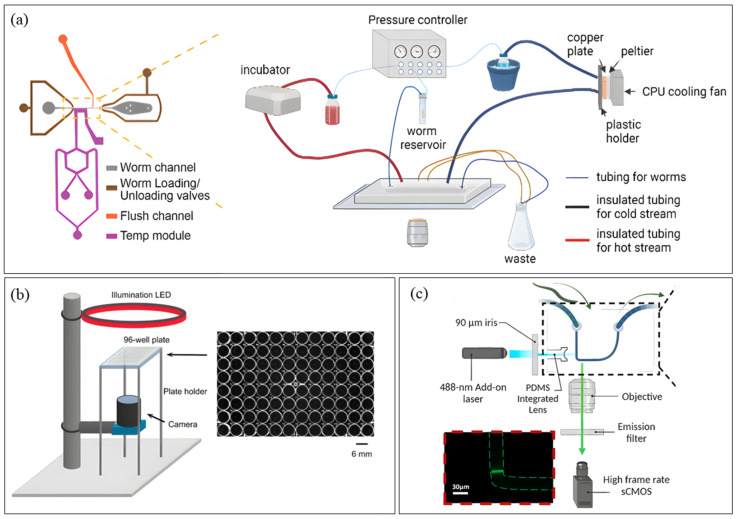
Devices for *C. elegans* Imaging. (**a**) Microfluidic Platform platform (Reproduced with permission from [Sol Ah Lee et al. [66]], [Biophysical Journal]; published by [Elsevier], [2024]); (**b**) Non-Microfluidic platform (Reproduced with permission from [Ji et al. [68]], [Genetics]; published by [Oxford University Press], [2024]); (**c**) Optofluidic platform (Reproduced with permission from [Faraz Rahimpouresfahani et al. [70]], [RSC Advances]; published by [Royal Society of Chemistry (RSC); RSC Publishing; Cold Spring Harbor Laboratory Press], [2024]). Panel (**a**) shows the overall setup of the dynamic temperature control system on the right and the detailed image of the microfluidic device on the left. The schematic layout of the imaging system with 96-well-plate containing individual worms in each well is depicted in panel (**b**), and panel (**c**) portrays the mechanical design of the optical components embedded into the optofluidic chip.

Following efforts to overcome single-worm capacity, worm orientation and motion artifacts in neural imaging, Berger et al. [65] developed a high-throughput microfluidic platform with 41 trap channels and hydraulic valves for immobilization and food supply. The platform enabled the first parallel and longitudinal live imaging of larval developmental stages, including vulval morphogenesis, using epifluorescence microscopy. The platform can perform in vivo RNAi assays and hints at the potential for gene expression quantification at high temporal resolution via fluorescent reporters. While allowing natural development with intermittent immobilization, imaging precision was compromised by partial immobilization, and multiple devices of different dimensions were required to accommodate all developmental stages. A single multilayer device with variable channel dimensions could simplify this workflow. Latest in the neural study sector, Lee et al. [66] integrated a flow channel containing two Peltier thermal control modules and an imaging channel into a microfluidic device (Figure 2a) for calcium imaging of temperature-induced neural response at single-neuron resolution. COMSOL (https://www.comsol.com/comsol-multiphysics, accessed on 7 July 2025) modeling was used to optimize flow and thermal parameters. The platform revealed sensitization in PVD neurons, where neurons reacted strongly to cold later after having a memory of the initial cold pulse, highlighting duration as an important variable in thermosensory circuit function. However, the platform required anesthetic-based immobilization, introducing potential artifacts into neuronal biology.

#### 3.1.2. Non-Microfluidic-Based Imaging Platforms

While microfluidic devices provide high-throughput with minimal operator handling and variability, their complexity can make them challenging for researchers to develop and implement. To balance automation with accessibility and reduce human error, many researchers prefer simpler arrangements such as multiwell plates or even Petri dishes equipped with integrated automation components. These formats are particularly advantageous for behavioral studies, since microfluidic confinement can sometimes alter worm behavior. In recent years, several such efforts have emerged in the worm imaging field for behavioral tracking. Puchalt et al. [67] engineered a Cartesian robot for a multiview imaging of worm motion in standard Petri dishes, preserving native behavior without microfluidic confinement. The setup consists of two microcameras in a laser-guided robotic head for software-based worm detection by high-resolution imaging, and two macrocameras for low-resolution imaging of the whole plate for mechanical worm tracking. They validated the platform via locomotion study, revealing significant motion differences between wild-type and mutant strains. The platform offered whole population tracking at once along with high-resolution single-worm observation, but tracking accuracy declined when worms interacted in crowded plates, an issue that could be addressed with deep learning models trained on worm morphology or by introducing worm-specific fluorescent markers. More recently, Ji et al. [68] advanced behavioral phenotyping by incorporating multiple behavioral states, like egg laying along with locomotion, with high-resolution, longitudinal and multimodal imaging of a 96 well-plate paired with computer vision (CV)-assisted behavioral analysis (Figure 2b). Their platform integrated dark-field illumination, CMOS imaging, and custom software (Mask-RCNN, MATLAB (https://www.mathworks.com/products/matlab.html, accessed on 22 July 2025), and Python (PyTorch GPU (v2.0.0 + cu1.1.8)) to acquire 20 s videos every 2 min over 5 h per assay. This system revealed that serotonin signaling aids locomotion decline necessary for egg laying and identified two previously unknown serotonin receptors, SER-1 and SER-7, involved in its regulation. The platform allowed simultaneous analysis of locomotion and egg-laying, while opening new avenues for rapid genetic and chemical screening, its CV algorithms showed reduced accuracy in egg counting within dense populations, suggesting the need for further model training on multiple experimental conditions with data from diversely populated eggs. 

#### 3.1.3. Optofluidic Platforms

While most *C. elegans* imaging platforms have relied on microfluidics aided with external microscopy arrangements, further advancement in this field introduced optofluidics, where optical components are directly embedded into microfluidic chips to create more compact, user-friendly and automated imaging systems. The recent works in this sector include Song et al.’s [69] ptychographic optofluidic chip, a lensless platform that combines coherent diffraction imaging (CDI) with flow cytometry for high-resolution imaging. The device features a microfluidic channel on the top layer and a scattering layer beneath that serves as a computational lens, enabling phase reconstruction to generate ptychographic images at video rates of ~30 frames per second. This platform addressed challenges with positional shifts and rotation angle detection, demonstrated potential for dynamic behavioral observation and slow-moving specimens with high resolution, and proved to reconstruct an image of similar quality as of regular microscope under both electrokinetic and pressure-driven flows. But the device is still constrained by restricted internal distortion or bending of worms and the inability to incorporate fluorescence imaging, with authors suggesting speckle illumination integration in this regard. In another work, Rahimpouresfahani et al. [70] developed a low-cost, PDMS-based optofluidic platform (Figure 2c) for high-throughput light-sheet imaging of transgenic worms using inverted fluorescence microscopy. The system comprised three key components: a flow delivery system, an imaging system containing a free-space laser diode, and an optofluidic chip integrating a lens microchannel for light-sheet generation and partial worm immobilization. Their study revealed low neuronal expressions under neurotoxic exposure and quantified aging-induced α-synuclein protein aggregation in ERS100 worms, a Parkinson’s disease model. Previous light-sheet systems could not image multiple developmental stages, but this platform allowed imaging at L3 and young adult stages, though embryonic-to-adult imaging was not achieved. Moreover, the device achieved a throughput of >20 worms/minute, though this was limited by the frame rate and exposure time.

Altogether, *C. elegans* imaging has advanced greatly towards high-throughput and single-animal resolution. A persistent trade-off remains between speed and resolution; higher throughput often reduces image detail, while higher resolution slows acquisition; yet this balance can be optimized according to the needs of each study. Achieving fully automated and bias-free imaging also introduces fabrication and operational complexity. But, once researchers overcome the initial challenges of device fabrication and training, these platforms offer long-term benefits in worm research.

## 4. Assay Automation and Aging Analysis

*C. elegans* has long been a leading model for aging research due to its short lifespan, transparency and ease of manipulation. Traditional plate-based aging assays, however, are labor-intensive, requiring daily or alternate-day worm transfers to fresh plates for progeny separation. This approach demands constant preparation of new plates and bacterial cultures, along with manual picking and scoring, which introduces a high workload and substantial variability. Errors can arise from inconsistent bacterial concentrations, handling damage during picking, or worms being lost by burrowing or crawling off plates. To minimize variability, manual labor and improve data reliability, researchers have developed automated assay platforms over time that support both high-throughput experimentation and detailed aging analysis.

### 4.1. Major Advances in Assay Automation Prior to 2020

One of the earliest comprehensive efforts for lifespan automation was Stroustrup et al.’s [71] Lifespan Machine, which employed flatbed scanners and image-processing software for automated worm scoring in a lifespan study, tracking survival curves under diverse environmental conditions. Churgin et al. [72] advanced longitudinal assays and imaging by a PDMS-based microfabricated platform with 240 wells, named WorMotel, allowing individual behavioral tracking over lifespan, highlighting the difference between lifespan and healthspan. Xian et al. [73] introduced WormFarm, a PDMS-based microfluidic system that fully automated long-term culture and real-time imaging of 30–50 worms individually with automated progeny removal and feeding, in contrast to WorMotel, which relied on 5-fluoro-2′-deoxyuridine (FUdR) to block progeny growth and lifetime bacterial supply instead of continuous feeding and progeny flushing. Similarly to the Lifespan Machine, WormScan [74] offered low-cost image-based survival scoring for phenotypic analysis rather than aging research with a simpler setup. Software-based efforts like CeleST [75] enabled high-content swim behavior analysis through quantitative kinematics and posture tracking.

These foundational systems reduced human intervention and improved experimental consistency, yet several technological and methodological limitations persisted. Key challenges included the lack of single-worm resolution for behavioral analysis, risk of misclassification of paralyzed but living animals as dead, leading to variability in lifespan quantification, potential risk of stress and toxicity induced by chemical agents like FUdR, and limited imaging resolution. To address these gaps, several next-generation automated assay platforms have recently been developed, as discussed below.

### 4.2. State of the Art in Assay Automation (2020–Present)

#### 4.2.1. Microfluidic-Based Platforms

Upscaling worm scoring and longitudinal worm assay through microfluidics, Rahman et al. [76] introduced the NemaLife chip with optimized micropillar, allowing natural worm crawling throughout their lifespan and mitigating the stress induced by chamber-specific swimming in liquid microfluidic environments. The chip eradicated manual worm transfer by reporting progeny flushing and feeding without any progeny-blocking drugs, but restricted full automation due to the operation being syringe-driven. By performing longevity analysis as well as RNAi knockdown, the chip is reported to perform reliable and efficient worm assays, scoring worm longevity and healthspan metrics (like pharyngeal pumping and locomotion traits), resolving the concern of reduced lifespan in microfluidic platforms. More on life-long worm assay, the microfluidic system HeALTH (Health and Lifespan Testing Hub) by Le et al. [77] provided precise spatiotemporal control in behavioral tracking of 60 worms per device. The device allowed continuous video recording with a motorized imaging stage, continuous flow via a custom pressure delivery system and Peltier-based temperature control. Notably, its application was limited to adulthood, as analysis commenced at the L4 stage excluding early developmental behaviors, but the platform opened new avenues for behavioral, aging and drug screening studies in tightly controlled environmental and diet conditions. The study revealed increased worm movement in dietary restriction, increased lifespan in high bacterial concentration and controlled diurnal temperature shifts. In another work, Krenger et al. [78] investigated the correlation of aging and metabolism via longitudinal oxygen consumption rate (OCR) measurements in a microfluidic system. The platform was fabricated using off-stoichiometry dual-cure thiol-ene-epoxy (OSTE+) polymer for its low gas permeability, enabling reliable OCR detection via luminescence-based oxygen sensing. The system confined and cultured worms from L4 through aging, automated feeding cycles with only single-worm handling. They unveiled that FCCP, a mitochondrial uncoupling agent, almost doubled OCR while azide collapses it, increased OCR from L4-aulthood, followed by aging-induced decrement. Worms experienced hypoxia-related developmental delays under long-term assays due to intermittent oxygen resupply, which can be addressed by either extending culture phases or reducing measurement phases. Statzer et al. [79] engineered a microfluidic platform to study the relationship between healthspan and sickspan in wild-type and long-lived mutants by assessing muscle strength and dynamic power. The system employed high frequency acoustophoretic force fields, generated by a piezoelectric transducer, to apply contactless and uniform mechanical challenges to swimming worms. This approach minimized behavioral variability inherent in movement-based assays and provided a direct readout of neuromuscular function. Their study revealed that muscle strength declines with age and that long-lived mutants exhibit proportional extensions of both healthspan and sickspan, rather than a selective compression of sickspan. A key limitation, however, is the platform’s low throughput, as measurements must be conducted on individual worms.

**Figure 3:**
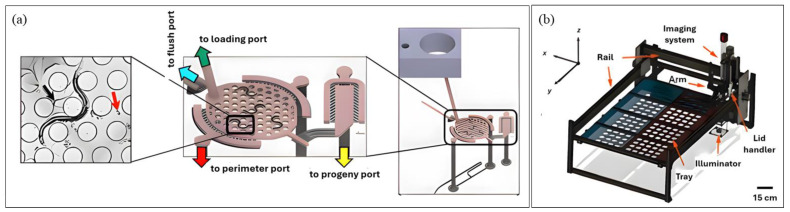
Devices for *C. elegans* Imaging (**a**) Microfluidic Platform (Reproduced with permission from [Sohrabi et al. [80]], [Lab on a Chip]; published by [Royal Society of Chemistry (RSC)], [2023]); (**b**) Non-Microfluidic platform (Reproduced with permission from [Li et al. [85]], [PNAS Nexus]; published by [Oxford University Press], [2023]). Panel (**a**) depicts the device CeLAb, where the left section shows natural worm movement through microchannels, the middle section shows multiple worms housed in the platform with a progeny port for progeny trapping, followed by the right section of the image giving an overview of the setup. Panel (**b**) depicts the overall WormPicker device.

Some notable works focusing on reproductive span have been introduced over the years. The two latest contributions in this regard are the CeLab platform developed by Sohrabi et al. [80] and the Egg-Counter platform by Banse et al. [81]. The CeLab is a reusable, plug-and-play, multilayer PDMS device (Figure 3a) containing 200 individual chambers that support simultaneous assays of lifespan, reproductive span and progeny production. The design incorporates hexagonally arranged microposts to promote natural crawling, integrated sieves for progeny flushing, on-chip valves for fluid handling and an embedded well-plate interface for efficient worm loading. CeLab achieved a ~6-fold increase in throughput compared to traditional plate assays and facilitated simultaneous, individual-level analysis of multiple life-history traits. The platform revealed that both lifespan and reproductive span are extended by low-dose metformin and heat-killed bacterial diets. Despite these advances, the system still required daily manual feeding and scoring, highlighting an area for future automation. Meanwhile, the Egg-Counter platform is solely focused on the egg-laying event. This microfluidic system accommodated 32 worms in hexagonal micropillar arenas that promote natural movement during 48 h experiments and incorporated independent perfusion lanes for side-by-side genotype comparisons. A centralized imaging zone captured all laid eggs across channels, while thermoelectric cooling maintained precise environmental conditions (±0.1–0.2 °C). Using this semi-automated platform, the authors characterized egg-laying dynamics under standard culture temperatures (15 °C and 20 °C), revealing temperature-dependent effects on reproductive behavior, which was previously unexplored.

#### 4.2.2. Non-Microfluidic-Based Platforms

Complementing microfluidic designs, another stream of engineering targeted assay automation standard culturing conditions. The earliest effort in this regard dates to 2021, when Puchalt et al. [82] engineered an active-vision imaging system, named SiVis, to automate lifespan scoring on standard Petri dishes by monitoring plate-cultured worms with illumination and motion detection-based image analysis. The device accommodates a pallet holding two Petri dishes, with ventilation for temperature regulation, bottom-mounted lighting, dual top-mounted cameras, and a closed-loop PID controller. Using this AI-incorporated platform, authors showed to have no significant difference between manual and automated lifespan scoring, validated the metformin effect on longevity, although reliance on motion detection occasionally led to false positives and negatives. Afterwards, Kerr et al. [83] developed ‘The *C. elegans* Observatory’, a large-scale robotic system integrating imaging and real-time analysis for longitudinal behavioral aging and lifespan studies. The platform housed 576 plates within a custom incubator with temperature and humidity control, running four simultaneous assays and imaging each plate four times per day. Its design incorporated brightfield imaging, two-axis robotic stages and solenoid-driven mechanical stimulation. Unlike SiVis, which was restricted to simultaneous observation of two plates, the Observatory achieved higher throughput, genome-wide RNAi screening, accurately capturing both lifespan and diverse behavioral phenotypes, thereby uncovering novel regulators of aging dynamics. The system lost individual worm identity upon worm interactions, which could be addressed in future upgrades. Recently, Zavagno et al. [84] attempted to study healthspan-associated aging, focusing solely on dynamic worm motion through their plate-based system named ‘WormGazer™’. The platform showed that most functional decline happens in week 1, and 7 days of movement carries most information of a 40-day lifespan assay, simplifying a 40-day lifespan assay to 7–14 days using a low-cost distributed camera array capable of imaging multiple Petri dishes under controlled temperature with real-time motion quantification. They reported knocking tank-1 gene to extend youthful behavioral performance without affecting lifespan and cdc-42 to cause premature behavioral decline, but failed to distinguish between motion phenotypes (e.g., tremor, paralysis, and hypoactivity). Collectively, these plate-based platforms retained native culturing conditions but were constrained by individual worm identity loss over worm interactions and the crowd.

Lastly, Li et al. [85] fabricated a robotic tool named ‘WormPicker’ (Figure 3b) to automate genetic studies. The system integrated a deep learning-based multi-DOF robotic arm for worm transfer on agar plates with a throughput of (~30–60 worms/min) and optical tools in one 3D motorized stage. Using machine vision, it identifies individual worms by size, sex, and fluorescence, can autonomously perform genetic crosses, mapping, and transgene integration. The system reduced manual labor drastically, but its one-at-a-time workflow limits throughput and increases experimental duration compared to population-level approaches.

## 5. High-Throughput Screening

*C. elegans* is particularly well-suited for HTS due to its low maintenance cost, ease of genetic and physiological manipulation, suitability for disease modeling and short lifespan. HTS enables large-scale data collection from extensive *C. elegans* cohorts by integrating micromanipulation techniques with advanced imaging platforms. HTS approaches can be broadly categorized into the following: chemical screens, which identify compounds with desired effects from large chemical libraries [8]; drug screens, which test numerous therapeutic candidates for disease-specific efficacy [86,87]; genetic screens, which involve gene knockdown or mutation to uncover phenotypes of interest [8]; aging screens, which leverages high-throughput behavioral and imaging assays to track lifespan and healthspan for aging biomarker identification [88]. As a result, HTS in *C. elegans* has facilitated the large-scale discovery of genes, chemical modifiers, biomarkers, and drug candidates over the years.

### 5.1. Notable Advances in HTS Platforms up to 2020

Conventional HTS methods relied on agar plate-based assays that required frequent worm transfers through manual picking, tightly scheduled handling and extensive labor. Imaging also depended on manual immobilization using anesthetics, which can introduce biological side effects. These approaches were prone to operator bias while requiring long time to acquire data. Such challenges have driven the development of recent automated platforms to improve efficiency and consistency. Some notable non-microfluidic HTS platforms include Kwok et al.’s [89] 24-well-plate based chemical screening platform identifying compounds affecting worm development, morphology and viability; Ashrafi et al.’s [90] large-scale RNAi screen revealing fat regulatory genes and pathways for normal fat metabolism; Hunt-Newbury et al.’s [91] genetic screening platform that built a library of gene expression pattern by screening ~ 1886 genes, and Petrascheck et al.’s [92] 384-well-plate based platform discovering a human antidepressant to have lifespan-extending effect in worms by screening 88,000 chemicals. Well-plate-based systems still require manual steps such as chemical addition, micromanipulation, worm loading and transfer. In contrast, microfluidic platforms integrate automated worm handling, micromanipulation (e.g., immobilization and sorting), precise liquid control and high-resolution imaging, all while handling large worm populations. Some prominent contributions in this area include Carr et al.’s [93] microfluidic worm chip integrated with locomotion-tracking software, designed for drug screening and assessing dose-dependent effects on locomotion using electrotaxis at single-worm resolution, while Mondal et al.’s [94] microfluidic device, inspired by 96-well-plates, enabled simultaneous high-resolution drug screening of ~4000 animals in just 16 min, identifying four confirmed modulators of polyglutamine aggregation. Samara et al. [95] created an integrated system combining femtosecond laser axotomy within a microfluidic platform and custom software for worm handling, transfer, imaging and microsurgery, allowing in vivo chemical screening that identified PKC inhibitors as modulators of neuronal regeneration. Prominent genetic screening platforms include Crane et al.’s [96] automated, computer vision-based microfluidic system for autonomous screening using morphometric features, discovering new mutants with previously uncharacterized genes and phenotypes involved in synaptogenesis and achieving throughputs up to 100 times faster than prior methods. Aging and developmental HTS systems exemplified by Letizia et al.’s [97] microfluidic chip with high-resolution imaging, conducted longitudinal phenotype tracking of individual worms throughout their life cycle under drug exposure, including at the embryonic stage.

Collectively, these platforms demonstrate how advances in micromanipulation and imaging technologies have transformed HTS, driving biological discoveries across genetics, drug testing, aging and neurobiology. However, challenges remain, including limited post-screening recovery, the need for multiple platforms to achieve true high throughput and partial automation. The following section examines recent systems designed to bridge these gaps.

### 5.2. Trends and Advances in HTS Platforms(2020–Present)

#### 5.2.1. Drug Screening and Behavioral Phenotyping

**Figure 4:**
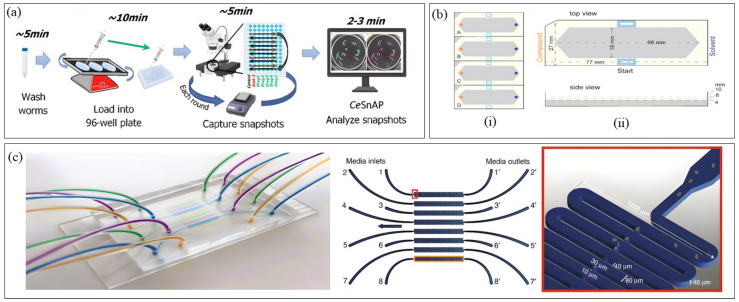
High-Throughput Screening platforms. (**a**) Drug Screening and Behavioral Phenotyping platform (Reproduced with permission from [Sohrabi et al. [98]], [Communications Biology]; published by [Springer Nature], [2021]); (**b**) Chemical Screening platform (Reproduced with permission from [Fryer et al. [101]], [PLOS Biology]; published by [PLOS], [2024]); (**c**) Developmental and Genetic Screening platform (Reproduced with permission from [Huseyin Baris Atakan et al. [102]], [microsystems & nanoengineering]; published by [Springer Nature], [2020]). Panel (**a**) shows the overview of the entire assay using *Ce*SnAP, with the approximate time required by the operator in each step. This platform sped up manual assay by 40 times. Panel (**b**) the schematic of the standard well-plate, where section (i) shows 4 lanes- A, B, C, D having inserted foam to confine worms and standardize chemotaxis assay arenas and section (ii) depicts single foam insert dimensions from top and side view. Panel (**c**) depicts the overview of the 8-laned microfluidic chip with inlet, outlet, and channel dimensions.

In the realm of neurobiology-focused high-throughput platforms, the *Ce*SnAP platform (Figure 4a) developed by Sohrabi et al. [98] pioneered a machine learning-based approach for drug screening and behavioral phenotyping. Focusing on the spasm-like “curling” motor phenotype induced by the RNAi knockdown of bcat-1, a gene associated with Parkinson’s disease (PD), the system conducted high-throughput screening of 17,000 worms to identify compounds that restore motor function. Using automated imaging in 96-well-plates and a novel CNN-based workflow for curling phenotyping, *Ce*SnAP screened 50 FDA-approved drugs, six times faster than a conventional assay, and identified four drug candidates with motor regeneration potential. By analyzing still snapshots instead of video, the platform significantly reduced data storage requirements and processing time, leveraging the platform’s application to therapeutic discovery. However, its reliance on static images limits the ability to capture dynamic behavioral phenotypes. Addressing this need, O’Brien et al. [99] developed a standardized, scalable assay for high-throughput behavioral phenotyping and drug repurposing screening in Mendelian disease models. Using CRISPR, they generated 25 *C. elegans* disease models and tracked their behavioral phenotypes in 96-well-plates by preparing the worms on agar plates, transferring to imaging plates via a COPAS 500 Flow Pilot, and recording video for CNN-based behavioral tracking. Afterwards, hierarchical clustering enabled comprehensive behavioral fingerprinting, extracting ~2763 features per assay, proving that functionally related genes yield similar phenotypes. Drug repurposing screens on unc-80 mutants, chosen for their distinct behavioral profile, identified previously unreported two compounds capable of restoring normal behavior. In contrast to *Ce*SnAP, which prioritized speed and simplicity through static snapshots, O’Brien et al.’s [99] platform emphasized behavioral depth by incorporating dynamic video-based analysis while maintaining high throughput and scalability. However, the therapeutic relevance of identified hit compounds remains to be validated for translatability.

#### 5.2.2. Chemical and Toxicity Screening

Dranchak et al. [100] conducted an in vivo quantitative high-throughput screening (qHTS) using GFP-labeled *C. elegans* to study response to anti-infectives and toxicants. Employing a 384-well-plate format with laser-scanning cytometry (LSC), they screened 643 anti-infectives and 887 environmental chemicals, overcoming the common issue of bacterial overgrowth by using heat-inactivated bacterial ghosts as an alternative food source. The platform also supported follow-up mechanistic studies, including life stage-specific effects, proteomics and comparative toxicology. While offering a rapid whole-organism screening workflow for assessing chemical impacts on worm phenotype and viability, it remains semi-automated due to manual worm handling, a limitation that could be mitigated with future automation. Additionally, LSC provides rapid plate reading but with limited phenotypic resolution compared to microscopy, which, if adopted, would improve detail but reduce throughput. Focusing on worm chemotaxis, Fryer et al. [101] introduced a multi-well-plate-based chemical screening platform (Figure 4b) to quantitatively assess the valence responses of plant-derived small molecules (SMs) in *C. elegans* with higher throughput and reproducibility than traditional assays. The system automated the manual chemotaxis workflow by integrating hardware for liquid and worm handling, Python-based OWL software for automated image analysis to count worms and chemotaxis strength/direction analysis, wetware for assay setup, and a flatbed scanner for imaging. The study discovered 37 chemoactive SMs, after screening 90 SMs, with 10 of them having multiple chemosensory neurons triggering the response valence, highlighting how olfactory valence is determined at the circuit level rather than by single receptors. While this approach offered greater scalability and efficiency than manual chemotaxis assays, it was limited to a single concentration per compound and a single time point, restricting the ability to detect concentration-dependent or time-dependent effects along with lack of examination of physiological influences on chemosensory behavior. Potential improvements include incorporating microfluidic systems for multi-concentration testing, real-time tracking for dynamic behavior analysis, and temporal control of stimulus delivery.

#### 5.2.3. Developmental and Genetic Screening

Recent advances in *C. elegans* HTS have expanded into previously underexplored areas such as embryogenesis and transgenesis. Atakan et al. [102] developed a multiplexed microfluidic platform (Figure 4c) for automated embryogenesis phenotyping under varying chemical and osmotic conditions, capable of tracking up to 800 embryos per chip. Using a custom MATLAB-based image processing pipeline with CNN segmentation, the system classified embryos into three developmental stages: bean, twitching, and hatching. Designed for simplicity and accessibility to non-engineering users, the chip relied on passive hydrodynamics for embryo loading and immobilization, avoiding the need for valves or actuators. The platform revealed that toxic doses of NaCl and glucose delayed development and increased lethality, whereas sucrose and glycerol had milder effects. Although variability in bleaching and bacterial concentration from manual bleaching, along with the absence of automated debris filtration, remain limitations, this easy-to-fabricate semi-automated platform establishes embryos as a novel alternative for high-throughput *C. elegans* studies under controlled osmotic conditions. In a different domain, Stevenson et al. [103] developed Transgenic Arrays Resulting in Diversity of Integrated Sequences (TARDIS), a high-throughput transgenesis platform capable of generating hundreds of genetically distinct lines from a single microinjection. The workflow operates in two phases: (1) introduction of a synthetic DNA library into the germline via traditional microinjection, producing stable heritable extrachromosomal arrays (TARDIS libraries) without immediate integration, and (2) subsequent Cas9 induction to extract and integrate individual library elements into engineered genomic landing pads. This two-step approach achieved an estimated ~1000-fold increase in throughput compared to single-copy CRISPR methods and supports barcode generation and promoter integration at multiple genomic loci. A key limitation is the reliance on skilled manual microinjection for library delivery, which could be addressed by incorporating automated microinjection systems as discussed previously.

All in all, recent HTS platforms relied mostly on multi-well-plates integrated with AI-driven approaches for advanced image processing.

For easy navigation of trade-offs across platforms, a comparative table is presented in Table 1 below:

## 6. Discussion and Future Prospects

From the early 2000s onward, engineering-driven platforms have progressively reduced variability, increased throughput, and improved data quality via automation in various aspects of worm assays. The mid-2000s experienced a rapid expansion in automation, which focused mostly on individual operations like immobilization-based, longitudinal culturing-based, imaging-based, or worm scoring-based devices. While these efforts proved feasibility, building a new chip for each individual function is neither scalable nor efficient, putting emphasis on shifting towards multimodal platforms capable of combining multiple operations into a single integrated device. Optofluidic chips, for example, demonstrated how microfluidics could be coupled with optical imaging to enable immobilization and visualization within the same platform [69,70].

In parallel, the rapid progress of AI has fundamentally reshaped *C. elegans* automation. Machine and deep learning approaches now perform tasks that once required expert human judgment, including automatic worm scoring [71,74,82,83], strain recognition [85,98,99], behavioral annotation [72,75,77,83,84,98,99,101], and healthspan quantification [72,76,77,78,79,80,84]. These AI-based pipelines not only reduce human bias but also allow complex datasets such as posture dynamics, pumping rates, or survival trajectories to be analyzed consistently across large populations. Looking ahead, a particularly promising direction is the development of closed-loop experimentation systems. Instead of static and preprogrammed protocols, closed-loop systems dynamically monitor experimental parameters and adjust them in real time. For example, if temperature drifts from its set point, flow rates deviate, or bacterial food levels become suboptimal, the platform could automatically detect and correct these changes. To add more to this, AI models can also be used to build custom software trained on optimized flow rates. If the system detects abnormal flow, the AI model can send a signal to the pump or flow controller to adjust from its current setting to the correct target flow rate. Such adaptive feedback will transform worm assays from passive observation into interactive, optimized experimentation. Thus, the next step can be embedding these additional modules, such as robotic arms [85] for automated worm picking and microinjection, multilayer transparent chips to simultaneously accommodate large cohorts of different strains and mutants for life-long *C. elegans* culturing and imaging, and AI-integrated software for automated image processing, worm visualization, and scoring, together unified within a single multimodal closed-loop platform.

Designing platforms that ensure not only automation but also reliable data generation and accuracy, environmental stability, and unbiased scoring is crucial, which in turn requires increasingly intricate fabrication processes that are non-trivial even for engineers, and far beyond the capacity of most biology laboratories. Building custom multimodal devices for each project is impractical, time-consuming, and diverts focus away from biological discovery to device construction. Scalability of these platforms is mostly hindered by either accessibility (especially for non-engineering labs), a costly fabrication process, or expensive maintenance. A summary table guiding through the recent platforms suitable for low-resource labs and high-throughput facilities is provided in Table 2 below:

A critical future step would be the commercialization of integrated worm platforms. Standardized, commercially available devices would enable biologists to adopt automation without the need to fabricate or engineer their own systems. While most of the works introduced in the last five years were mostly academic, some platforms hold strong potential for commercialization, such as CeLab [80], Egg-counter [81], SiViS [82], *Ce*SnAP [98], Behavioral phenotyping and drug repurpose screening platform by O’Brien et al. [99], microfluidic platform for embryo phenotyping by Atakan et al. [102], and TARDIS [103]. Equally important is the development of unified software environments. Currently, multiple separate software tools are often needed, such as one for microscope imaging, another for flow control, and another for data analysis, making the workflow fragmented and difficult to master. A dedicated platform that integrates all components, from microfluidic and robotic control to imaging and analysis, within a single user-friendly interface would dramatically reduce the learning curve and enable easy operation. Such systems would empower researchers from non-engineering backgrounds to perform sophisticated, high-throughput experiments using a single software environment. Some of the strong candidates for multimodal system might be combining CeLab [80], Egg-counter [81] and SiViS [82], which altogether would cover lifespan, reproductive span and brood size assays in one platform; for sophisticated behavioral phenotyping *Ce*SnAP [98], behavioral phenotyping platform by O’Brien et al. [99] and Ji et al.’s [68] 96 well-plate multimodal imaging platform can be merged; study leveraging embryogenesis to larval developmental stages could be advanced by combining Berger et al.’s [65] microfluidic imaging method with Atakan et al.’s [102] embryo phenotyping microfluidic platform; Pan et al.’s [53] robotic microinjection platform could be merged with TARDIS [103] to develop an automated transgenesis platform, resolving manual microinjection trade-off of TARDIS.

Lastly, ethical considerations should not be overlooked when working with *C. elegans*. Although this invertebrate model is not subject to the same regulations as vertebrates, minimizing unnecessary stress remains important for both welfare and scientific integrity. Automated platforms should therefore be designed to mimic natural living conditions as closely as possible, with attention to environmental factors that influence worm health. In addition, manual handling steps such as worm picking and transferring should be performed carefully to avoid mishandling or accidental mortality.

In summary, while the past two decades established the foundation for *C. elegans* automation, the past five years have shifted the focus toward integration, intelligence, and adaptability. The field is moving beyond single-function chips toward multimodal, AI-driven, closed-loop platforms. Commercialization and software unification will be key to ensuring that these technologies are not limited to specialized labs but become widely accessible tools that can accelerate biological discovery across the *C. elegans* community.

## 7. Concluding Remarks

In the last decade, *C. elegans* research has been transformed by miniaturization, enabling tightly controlled experimental conditions and higher throughput. Microfluidics, in particular, became central across nearly all worm assays, providing precise environmental control and reducing variability. However, innovations of the past five years have shifted toward a new frontier, integrating AI and robotics into these miniaturized platforms. While microfluidic devices and multi-well-plates could culture, manipulate, and maintain precise environmental control for large worm populations, data analysis, like scoring lifespan, healthspan, motility, or developmental stage, still relied heavily on researcher judgment. Recent advances in machine learning and deep learning now automate this step by analyzing images and videos to classify traits, detect abnormalities, recognize strains, and perform lifespan scoring. Moreover, although microfluidic platforms and multi-well plates already reduced experimental variability and automated many aspects of worm assays, several critical steps, like worm picking, transferring, and precise worm positioning, remained manual. In recent times, robotics and automated actuators have begun addressing these manual bottlenecks, like a robotic arm for worm picking [85], robotics for microinjection [53], micropositioner for worm positioning [50], etc. If these diverse developments can eventually be merged into a single multimodal platform, they would bring about a revolutionary acceleration in *C. elegans* experimentation, propelling biological research into a new, faster, and more reproducible dimension.

## Figures and Tables

**Table 1:** Comparative summary of trade-offs across automated platforms in *C. elegans* research across major functional categories.

Platforms	Functional Category	Throughput	Resolution	Cost	Automation Level
SAW-based microfluidic platform [30]	Immobilization	Low (1 worm at a time)	Subcellular	Low	Semi-automated
Copli [31]		High (~1.5–9 worms/min)	Submicron (0.5 μm transverse and ~4 μm axial)	Low	Semi-automated
Immobilization by hydrogel polymerization in microfluidic platform [32]		High (exact number not mentioned)	Cellular (10× widefield)	Moderate (>manual immobilization, <commercial microfluidic systems)	Semi-automated
COPAS Infinity [40]	Sorting	High (exact number not mentioned)	Multi-parameter detection (not imaging)	High	Fully- automated
Spiral microfluidic device [41]		10 embryos/min	Single-embryo	Low	Partially automated
3D printed microfluidic worm sorting device [42]		~100 worms/min	Subcellular	Low to Moderate	Semi-automated
Microfluidic microinjector for passive immobilization [50]	Microinjection & Microsurgery	~30 s/worm	Cellular (gonad-level)	Low to Moderate	Semi-automated
Paintbrush for worm handling [51]		at least 15 worms in <1 min	Subcellular	Very low	Manual
Poker chip [52]		2.32 min/worm	Subcellular	Low	Semi-automated
Robotic microinjection [53]		44.5 s/worm	Subcellular	Moderate To High	Highly automated
Ytterbium-doped fiber femtosecond laser for microsurgery [54]		single worm at a time	Cellular and subcellular (~1.3–1.6 μm)	Moderate To High	Manual
Thermal laser ablation [55]		Low (single worm at a time, few worms/hour)	Tunable, ~2–5 µm (single-cell to nerve cord)	Moderate	Manual
Femtosecond laser microdissection [56]		~5 neurons/hour	Submicron ablation precision	High	Manual
Rotatable microfluidic device for bilateral chemosensory neuron imaging [63]	Imaging	~10 worms/hour	Cellular resolution for bilateral neurons	Low To Moderate	Semi-automated
Long-term imaging microfluidic device for mitochondrial density tracking [64]		long-term single-worm imaging	Subcellular	Low	Semi-automated
Microfluidic imaging method for parallel live imaging of larval development [65]		~ 10 worms over 48 h	Single-cell (0.2–0.5 µm z-spacing with 40×–60× magnification)	Low	Semi-automated
Microfluidic imaging platform for in vivo imaging in dynamic temperature control [66]		single-worm calcium imaging	Single-neuron, sub-second temporal resolution	Low To Moderate	Semi-automated
Cartesian robot-based imaging with multiview motion tracking [67]		~90 worms per run	Worm-level and posture-level motion tracking (up to ~1 µm/pixel)	Moderate	Fully- automated
Computer-vision integrated multimodal imaging in multi-well-plates [68]		High (96 worms simultaneously with 5 h experiment time)	Organism-level behavioral resolution (18 µm/pixel)	Moderate	Fully- automated
Optofluidic ptychography [69]		~1000 cells in 5 min	Submicron resolution	Low To Moderate	Semi-automated to automated
Optofluidic device for light-sheet imaging [70]		~20–25 worms/min	Cellular-resolution imaging of dopaminergic neurons (lateral resolution 1.1 µm, axial 2.4 µm)	Low	Semi-automated
NemaLife chip [76]	Assay automation	~100 animals per chip	Single-worm level	Low	Semi-automated
HeALTH (microfluidic platform) [77]		60 worms/device	Organism-level behavioral resolution	Moderate	Highly automated
Microfluidic system integrated with luminescence-based oxygen sensing [78]		1 worm/chip	Metabolic resolution (oxygen consumption rate (OCR) in pmol/min per worm)	Low To Moderate	Semi-automated
Microfluidic device integrated with acoustophoretic force fields [79]			Physiological resolution (functional readouts-force/power)	Moderate	Semi-automated
CeLab [80]		~1000 worms/experiment, in 5 chips simultaneously	Organism-level (individual worm resolution)	Low To Moderate	Semi-automated
Egg-counter [81]		32 worms/chip	Behavioral and temporal	Low	Semi-automated
SiViS [82]		10–15 worms/55 mm plate	Organism-level (30 µm/pixel)	Low	Highly automated
*C. elegans* Observatory [83]		~40–60 worm/6 cm plate, up to 576 plates/incubator cycle	Worm-level (40 µm/pixel, morphology and behavioral metrics)	Moderate To High	Highly automated
WormGazer [84]		Images every 5 min	Population- and organism-level (10 µm/s movement threshold)	Moderate	Highly automated
WormPicker [85]		Sorts worms at ~3.2 ± 0.7 worms/min, 144 agar plates simultaneously	Organismal + cellular-level fluorescence phenotyping (dual magnification)	High	Highly automated
*Ce*SnAP [98]	High-throughput screening (HTS)	1800 snapshots/hr, 70 wells imaged in ~20 min. high	Organism-level	Low To Moderate	Highly automated
Behavioral phenotyping and drug repurpose screening using CRISPR [99]		High (thousands of worms/day, fixed number not mentioned)	12.4 µm/pixel at 25 fps	Moderate	Highly automated
Quantitative high-throughput screening using laser-scanning cytometry [100]		High (tens of thousands of worms/day, fixed number not mentioned)	Organism-level	Moderate To High	Highly automated
Multi well-plate-based behavioral screening platform for measuring chemotaxis [101]		hundreds of worms per run (fixed number not mentioned)	Population-level	Low	Semi-automated
Microfluidic platform for embryo phenotyping [102]		High (800 embryos/chip)	Single-embryo, 20× brightfield	Low To Moderate	Semi-automated
TARDIS [103]		hundreds–thousands of transgenics per run (fixed number not mentioned)	genome/sequence level	Moderate	Low

**Table 2:** Platforms suitable for low-resource labs and high-throughput facilities.

Platforms	Functional Category	Low-Resource Lab	High-Throughput Facilities
SAW-based microfluidic platform [30]	Immobilization	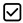	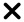
Copli [31]		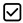	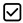
Immobilization by hydrogel polymerization in microfluidic platform [32]		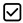	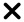
COPAS Infinity [40]	Sorting	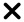	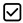
Spiral microfluidic device [41]		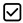	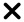
3D printed microfluidic worm sorting device [42]		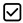	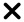
Microfluidic microinjector for passive immobilization [50]	Microinjection & Microsurgery	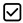	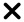
Paintbrush for worm handling [51]		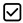	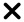
Poker chip [52]		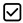	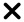
Robotic microinjection [53]		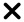	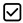
Ytterbium-doped fiber femtosecond laser for microsurgery [54]		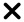	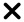
Thermal laser ablation [55]		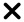	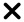
Femtosecond laser microdissection [56]		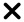	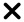
Rotatable microfluidic device for bilateral chemosensory neuron imaging [63]	Imaging	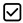	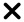
Long-term imaging microfluidic device for mitochondrial density tracking [64]		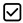	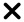
Microfluidic imaging method for parallel live imaging of larval development [65]		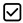	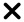
Microfluidic imaging platform for in vivo imaging in dynamic temperature control [66]		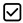	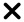
Cartesian robot-based imaging with multiview motion tracking [67]		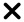	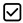
Computer-vision integrated multimodal imaging in multi well-plates [68]		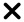	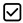
Optofluidic ptychography [69]		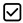	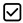
Optofluidic device for light-sheet imaging [70]		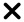	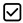
NemaLife chip [76]	Assay automation	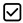	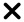
HeALTH (microfluidic platform) [77]		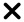	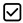
Microfluidic system integrated with luminescence-based oxygen sensing [78]		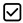	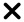
Microfluidic device integrated with acoustophoretic force fields [79]		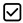	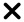
CeLab [80]		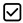	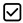
Egg-counter [81]		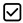	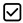
SiViS [82]		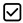	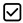
*C. elegans* Observatory [83]		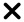	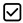
WormGazer [84]		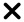	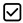
WormPicker [85]		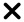	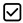
***Ce***SnAP [98]	High-throughput screening (HTS)	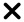	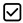
Behavioral phenotyping and drug repurpose screening using CRISPR [99]		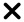	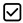
Quantitative high-throughput screening using laser-scanning cytometry [100]		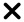	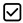
Multi well-plate-based behavioral screening platform for measuring chemotaxis [101]		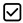	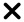
Microfluidic platform for embryo phenotyping [102]		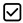	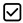
TARDIS [103]		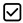	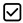

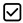
 indicates Suitable, 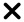 indicates Unsuitable

## Data Availability

No new data were created or analyzed in this study.
